# Postmortem diagnosis of COVID-19: Antemortem challenges of three cases at the 37 Military Hospital, Accra, Ghana

**DOI:** 10.4102/ajlm.v9i1.1290

**Published:** 2020-11-03

**Authors:** Seth A. Attoh, Frederick Hobenu, Lawrence Edusei, Kwasi Agyeman-Bediako, Clement T. Laryea, Edward O. Nyarko, Michael K. Amedi, Richard H. Asmah, Edward Asumanu, Mary McAddy, Anthony Maison, Godwin Nyarko, Raymond D. Fatchu, Kafui Akakpo

**Affiliations:** 1 J.M. Wadhwani Department of Anatomical Pathology, 37 Military Hospital, Accra, Ghana; 2 Department of Pathology, Korle-bu Teaching Hospital, Accra, Ghana; 3 Department of Medicine, 37 Military Hospital, Accra, Ghana; 4 Public Health Division, 37 Military Hospital, Accra, Ghana; 5 Department of Radiology, 37 Military Hospital, Accra, Ghana; 6 Department of Molecular Biology, University of Health and Allied Sciences, Ho, Ghana; 7 Department of Surgery, 37 Military Hospital, Accra, Ghana; 8 Department of Pathology, 37 Military Hospital, Accra, Ghana; 9 Department of Pathology, University of Cape Coast, Cape Coast, Ghana

**Keywords:** COVID-19, autopsy, postmortem diagnosis, false-negative, Ghana

## Abstract

**Background:**

Consistency among clinical symptoms, laboratory results and autopsy findings can be a quality measure in the diagnosis of coronavirus disease 2019 (COVID-19). There have been classic clinical cases that have met the case definition of COVID-19 but real-time reverse-transcription polymerase chain reaction (rRT-PCR) tests of nasopharyngeal swabs were negative.

**Objectives:**

This study aimed to share pathological observations of autopsies performed at the 37 Military Hospital’s Department of Anatomical Pathology on three presumed COVID-19 cases in Accra, Ghana.

**Method:**

Complete autopsies with detailed gross and histopathological analysis were conducted between April 2020 and May 2020 on three suspected COVID-19 cases, of which two had initial negative (rRT-PCR) nasopharyngeal tests. Postmortem bronchopulmonary samples of two cases were collected and tested by rRT-PCR for severe acute respiratory syndrome coronavirus 2 (SARS-CoV-2).

**Results:**

The two postmortem bronchopulmonary samples tested for SARS-CoV-2 by rRT-PCR were positive. Though no postmortem bronchopulmonary sample was taken from the third case, a close contact tested positive for SARS-CoV-2 in later contact tracing. For all three cases, lung histopathological findings were consistent with Acute Respiratory Distress Syndrome.

**Conclusion:**

The outcome of COVID-19 testing is dependent on the sample type and accuracy of sampling amongst other factors. Histopathological findings vary and may be dependent on a patient’s modifying factors, as well as the duration of infection. More autopsies are required to fully understand the pathogenesis of this disease in Ghanaians.

## Introduction

The global estimate of confirmed cases of coronavirus disease 2019 (COVID-19) as of 27 May 2020 stood at over 5.5 million in approximately 213 countries and territories with over 349 190 deaths, giving a mortality rate of 15.7%.^1^ In Ghana, the first confirmed COVID-19 case was reported on 12 March 2020. As at the end of May 2020, over 7303 cases, 34 deaths and 2412 recoveries had been recorded.^2^ COVID-19 emerged from Wuhan, Hubei Province, China in December 2019, and is clinically associated with viral pneumonia.^3,4^ Clinical, laboratory and radiological features for COVID-19 are non-specific; features are similar to other respiratory tract infections.^5^ Thus, mild symptoms of COVID-19 such as fever, cough, dyspnoea, myalgia and fatigue were initially treated and managed as pneumonia symptoms by healthcare workers.

The World Health Orginization (WHO) Situation Report 94 defines a suspected COVID-19 case as:

[*A*] person presenting with acute respiratory illness (fever and at least one sign/symptom of respiratory disease, e.g. cough, shortness of breath) AND no aetiology that fully explains the clinical presentation; OR A patient with an acute respiratory illness AND has been in contact with a confirmed or probable case of COVID-19 in the last 14 days before the onset of symptoms; OR A patient with severe acute respiratory illness (fever and at least one sign/symptom of respiratory disease, e.g. cough shortness of breath; AND requiring hospitalization) AND in the absence of an alternative diagnosis that fully explains the clinical presentation. (pp. 11, 12)

The clinical presentation of COVID-19 infection varies from mild to moderate to severe. The severe presentation is reportedly characterized by Acute Respiratory Distress Syndrome. In line with this, diffuse alveolar damage (DAD) is a reported postmortem pathological feature of COVID-19; DAD is the histological correlate of Acute Respiratory Distress Syndrome. In the acute stage, DAD is characterized by hyaline membrane formation in the alveoli and the organizing stage by interstitial widening due to oedema and fibroblast proliferation.^7^ However, there is an instance where DAD was not reported.^8^ Other pathological features that have been reported in the lung include: oedema, fibrinous exudates, reactive hyperplasia limited to some type II pneumocytes and patchy inflammatory changes with scattered multinucleated giant cells.^7^ Others report pathological similarities between these coronavirus infections: COVID-19, Severe Acute Respiratory Syndrome (SARS) and Middle Eastern respiratory syndrome.^3^

Though other pathological features have been reported in other organs, including the liver and heart, the changes in these organs have been less significant than those reported in the lung. The prominent role of endotheliitis in COVID-19 infection was highlighted in one such publication that reported the presence of viral inclusion in endothelial cells.^9^ The authors suggested that infection with SARS coronavirus 2 (SARS-CoV-2) results in endotheliitis in several organs from the involvement of the virus and as part of the host inflammatory response. The authors hypothesized that a strategy that targets endotheliitis in susceptible patients (people with diabetes, hypertension, obesity) is likely to improve survival in such patients.^9^

Among nucleic acid tests, the polymerase chain reaction (PCR) method is considered the ‘gold standard’ for the detection of COVID-19, and it is characterized by rapid detection, high sensitivity and high specificity.^10^ The specificity of most real-time reverse-transcription (rRT) PCR tests is estimated at 100%, because the design of the primers is specific for the genome sequence of SARS-CoV-2. However, false-negative reports may occur and have been associated with intended and non-intended activities during case detection, patient preparation, sample collection, packaging, storage, transport and reporting.^11^

The nasopharyngeal swabs or other upper respiratory tract specimens, including throat swabs or, more recently, saliva are the commonly used specimens for rRT-PCR diagnosis of COVID-19.^11^ Howbeit, the positivity rates of these samples vary; bronchoalveolar lavage has the highest positivity rate. In one study of 205 confirmed COVID-19 infections, the rRT-PCR positivity rate of bronchoalveolar lavage, sputum, nasal and oropharyngeal specimens were 93%, 72%, 63% and 32%, respectively.^3^

Although confirmed deaths due to COVID-19 are being recorded, autopsy findings of COVID-19 reported deaths in Africa, including Ghana, are largely unavailable. This report shares pathological observations of autopsies performed at the 37 Military Hospital’s J.M. Wadhwani Department of Anatomical Pathology on three presumed COVID-19 cases. It also highlights the challenges associated with managing presumed cases.

## Methods

### Ethical considerations

Due to the emergency nature of the pandemic, ethical review and approval was not conducted. However, all identifiers were removed from the report and verbal consent was also sought from the relations of the deceased, where required.

### Study site

The 37 Military Hospital, a 600-bed tertiary facility, is one of the largest hospitals in Ghana. Annually, the J.M. Wadhwani Department of Anatomical Pathology of the hospital conducts approximately 1500 hospital, medico-legal or coroner’s autopsies in its standard morgue facility.

The notices of death were received by the Department of Anatomical Pathology for two presumed COVID-19 cases who were being managed within the 37 Military Hospital. Also received at the department was a case referred from a peripheral hospital for a medico-legal or coroner’s autopsy to determine the cause of death. All cases were received between April 2020 and May 2020 and met the clinical case definition of COVID-19; antemortem SARS-CoV-2 tests of the two tested cases were negative. All cases were transported to the morgue under COVID-19 recommended protocols as published by the WHO.^12^

A complete autopsy was conducted on all cases with detailed gross and histopathological analysis. Examinations were performed in the department’s state-of-the-art morgue following guidelines for performing autopsies on presumed COVID-19 cases. Except in the coroner’s autopsy case, postmortem bronchopulmonary samples were collected, immediately placed in viral transport media and sent to the Noguchi Memorial Institute for Medical Research where rRT-PCR was performed.

Selected organs (lungs, heart, brain, kidneys, liver, spleen) were sampled and fixed in 10% buffered formalin for histopathological studies. Organ slides were made, stained with haematoxylin and eosin and examined by certified histopathologists. Tissue sections were retained in formalin and blocks appropriately stored. Findings from the autopsy were corroborated by both clinical presentations and laboratory outcomes.

## Results

### Case 1

The first case was a man with hypertension and diabetes who was in his late thirties. He had a non-productive cough, fever (39.5 °C) and had experienced breathlessness for six days before hospital admission. He alleged that he had no contact with a confirmed or probable COVID-19 case. A provisional diagnosis of COVID-19 was made, the patient was isolated, and a nasopharyngeal swab was taken for SARS-CoV-2 testing. The SARS-CoV-2 test result came in after his demise and was negative. Therefore, a coroner’s autopsy was requested and was performed to find out the cause of death (Table 1). No postmortem bronchopulmonary specimen was taken. However, following contact tracing, it was discovered that he had prior contact with a confirmed COVID-19 patient and that one of his caregivers tested positive for SARS-CoV-2.

**TABLE 1:** Autopsy findings of three presumed COVID-19 cases at the 37 Military Hospital, Accra, Ghana, April 2020 to May 2020.

Body system	Case 1: 38 year old male	Case 2: 60 year old female	Case 3: 55 year old male
External examination	Poorly preserved Congested conjunctiva Not pallor of mucous membranes, not jaundiced, centrally cyanosed Epidermal sloughing Greenish discolouration of the dermis No bi-pedal pitting oedema	Morbidly obese Not pallor of mucous membranes, not jaundiced Congested conjunctiva Male hair distribution Striae on abdomen, chest and upper thighs Central cyanosis No bi-pedal pitting oedema	Not pallor of mucous membranes, not jaundiced Central cyanosis No bi-pedal pitting oedema
Cardiovascular system	Heart enlarged (490 g) Concentric hypertrophy of left ventricle Moderate aortic atherosclerosis Normal coronary arteries	Heart enlarged (550 g) Left ventricular hypertrophy and mild dilatation Moderate atherosclerosis of aorta and coronary arteries	Heart massively enlarged (600 g) Left ventricular hypertrophy Moderate coronary and aortic atherosclerosis
Respiratory system	Lung (R – 790 g, L – 650 g) oedematous, congested and firm Focal areas of red hepatization Larynx, trachea and bronchi contain frothy secretions Pulmonary arteries, few fatty streaks	Lung (R – 820 g, L – 770 g) Petechial haemorrhages on pleural surfaces Severely congested, firm with mild oedema lung apices Bilateral pulmonary thrombo-embolism at the openings of the pulmonary arteries Frothy mucoid secretions of larynx, trachea, bronchi	Lung (R – 810 g, L – 760 g) Severely congested, moderately edematous lungs Frothy secretions of larynx, trachea and bronchi Hyperemic mucosa Atheromatous plaques in pulmonary arteries
Genitourinary system	Kidneys (R – 145 g, L – 155 g) No significant macroscopic abnormality	Kidneys (R – 155 g, L – 160 g) No significant macroscopic abnormality	Kidneys (R – 150 g, L – 160 g) Good cortico-medullary differentiation Normal calyces, pelves, ureters, bladder Enlarged nodular prostate
Gastrointestinal system	No significant macroscopic abnormality	Normal oesophagus Non-bleeding posterior duodenal ulcer Normal intestines (small and large)	No significant macroscopic abnormality
Hepatobiliary system and pancreas	Liver (1450 g) Fatty parenchyma Gall bladder and pancreas appear grossly normal	Liver (1480 g) Moderately fatty parenchyma Gall bladder and pancreas appeared grossly normal	Liver (1400 g) Mildly fatty parenchyma Gall bladder and pancreas appeared grossly normal
Central nervous system	Brain weight (1350 g).		Moderate atherosclerosis at circle of Willis Small infarct in right basal ganglia
Musculoskeletal system	No significant macroscopic abnormality	Morbidly obese	No significant macroscopic abnormality
Reticulo-endothelial system	No significant macroscopic abnormality	No significant macroscopic abnormality	Spleen (250 g) Normal intra-abdominal lymph nodes
Endocrine system	Normal thyroid, pituitary and adrenals	Normal thyroid, pituitary and adrenals	Normal thyroid, pituitary and adrenals
Molecular	No rRT-PCR done	Positive for SARS-CoV-2	Positive for SARS-CoV-2

L, left; R, right; rRT-PCR, real-time reverse-transcription polymerase chain reaction; SARS-CoV-2, severe acute respiratory syndrome coronavirus 2.

**FIGURE 1:**
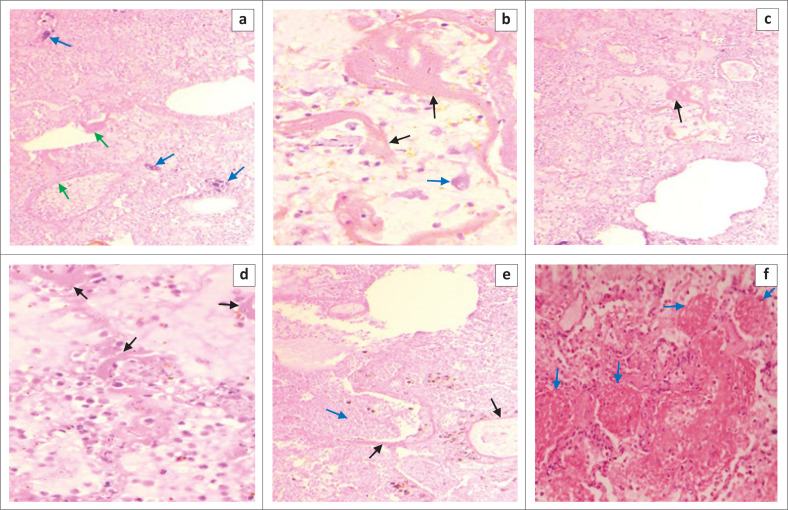
Haematoxylin and eosin staining of lung tissue samples from a 38 year old male patient presumed to have COVID-19 (37 Military Hospital, Accra, Ghana, April 2020). Case 1: (a) Autolytic changes with bacterial colonization in the smaller airways (blue arrows) ×100. Diffuse alveolar damage is noted with hyaline membrane formation (green arrows) ×100. (b) Higher magnification showing hyaline membrane (black arrows) and alveolar macrophages (blue arrow) ×400. (c) Diffuse alveolar damage with prominent hyaline membrane formation (black arrow) ×100. (d) Hyaline membrane formation (black arrows) ×400. (e) Pneumonic changes with mixed inflammatory cells exudate (blue arrow). Hyaline membrane (black arrow) ×100. (f) Area of microthrombi in smaller pulmonary capillaries ×400.

Microscopic sections of the lungs show autolytic changes with bacterial colonization of the bronchioles (Figure 1). The prominent pathological findings in fairly preserved areas of the lungs were severe oedema and DAD with prominent hyaline membrane formation, infiltration of the interstitium by macrophages and scattered multinucleated giant cells. Also, there was evidence of pneumonic changes in the lungs: moderate dense inflammatory cell exudate. In the final autopsy report, the cause of death was listed as bronchopneumonia most likely due to or as a consequence of COVID-19; diabetes and hypertension were contributory causes.

### Case 2

The second case was a 60-year-old woman with hypertension who was morbidly obese and had in the past week been diagnosed with diabetes mellitus. She had a three-week history of worsening breathing difficulty, one day of unproductive cough and no history of asthma. She allegedly had no contact with a confirmed or probable case of COVID-19. She was being managed for bronchitis or asthma with no improvement. An initial diagnosis of pulmonary thromboembolism was made. Differential diagnoses were congestive cardiac failure due to hypertension or COVID-19 infection. She was therefore transferred from the emergency centre to the isolation unit. She was managed with intravenous fluids, anticoagulants, antibiotics, insulin and anti-hypertensive medications. A nasopharyngeal swab was taken for a COVID-19 test three days after admission.

She experienced palpitation and dyspnoea but no orthopnoea, paroxysmal nocturnal dyspnoea, pedal oedema, calf tenderness or fever. She had an oxygen saturation of 85% at room air and 95% at non-rebreather oxygen, a normal pulse and a blood pressure of less than or equal to 140/90 mmHg throughout admission.

**FIGURE 2:**
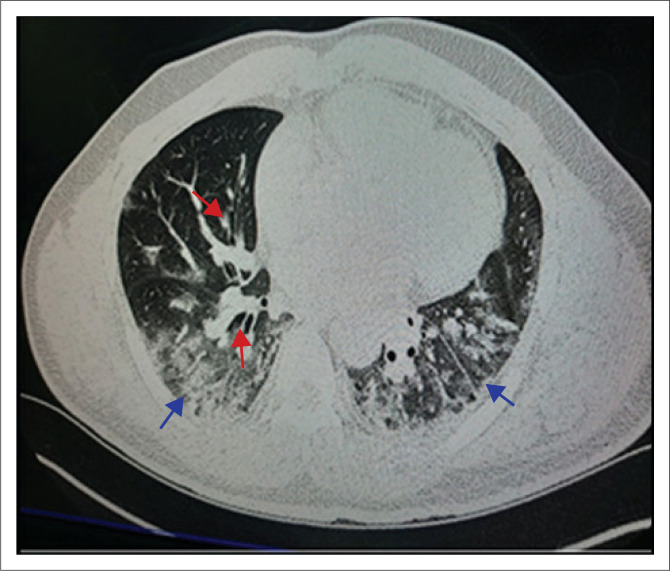
High resolution computerised tomography scan of a 60 year old female patient with COVID-19 (37 Military Hospital, Accra, Ghana, April 2020). Case 2: Axial image showing traction bronchiectasis in an area of ground-glass opacities (red arrows) and bilateral ‘crazy paving’ opacities (blue arrows).

She had a marginally increased neutrophil-white blood cell count and a high D-dimer of 1.26 ug/mL (fibrinogen equivalent unit; normal limit 0.0–0.5). Her liver function test showed deranged liver enzymes. However, renal function tests, C-reactive protein (3.6) and troponin were normal. A computerised tomogram scan was done, the pulmonary vessels were reported as normal (Figure 2).

The PCR results for COVID-19 were returned as negative three days after sample collection. She was therefore transferred from the isolation unit to a general medical ward. She later suddenly developed severe respiratory distress, started desaturating and later died. An academic autopsy was ordered by the attending clinical team who queried the PCR COVID-19 result (Table 1).

**FIGURE 3:**
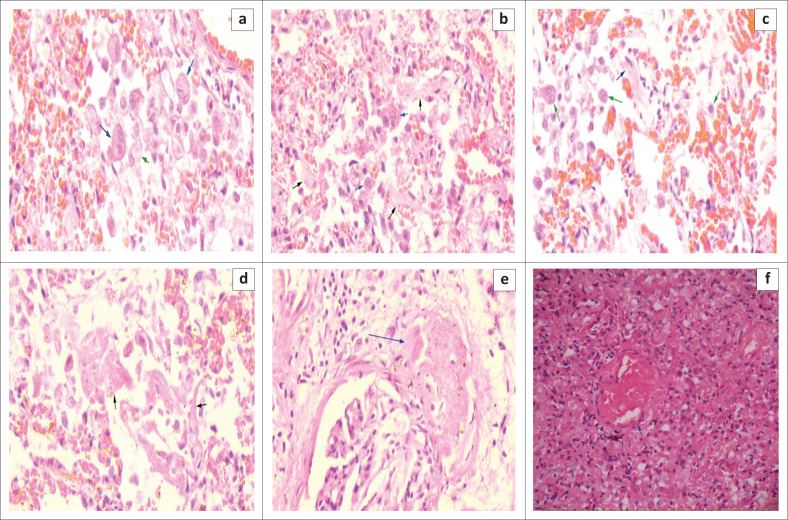
Haematoxylin and eosin staining of lung tissue samples from a 60 year old female patient with COVID-19 (37 Military Hospital, Accra, Ghana, April 2020). Case 2: (a) Alveolar sacs filled with alveolar macrophages (green arrow) and multinucleated giant cells (blue arrow) ×400. (b) Fibrous tissue proliferation in alveolar sacs (black arrow). Also noted are alveolar macrophages (blue arrow) ×400. (b) Fibroblast proliferation (green arrow) ×400. (d) Diffuse alveolar damage with prominent hyaline membrane formation (black arrow) x400. (e) Thrombus at the glomerulus (blue arrow) ×400. (f) Thrombus in a small pulmonary artery (blue arrow) ×100.

A postmortem bilateral lung parenchymal swab was taken for SARS-CoV-2 testing. Microscopic examination of the lungs showed severe congestion with foci of haemorrhage. There was a proliferation of fibroblasts and infiltration of macrophages within the interstitium and in the alveolar space; DAD with characteristic hyaline membranes in the alveoli; and interstitial fibrosis and oedema. Fibrin thrombi, mostly located in the subpleural region were noted. The liver and spleen were poorly preserved. Microthrombi were also noted in some of the glomeruli (Figure 3). In the final autopsy report, the cause of death was listed as acute pulmonary embolism due to or as a consequence of COVID-19 pneumonia; diabetes mellitus and hypertensive heart disease were contributing causes.

### Case 3

The third case was a 55 year old man with hypertension and diabetes who had a seven-day history of non-productive cough and dyspnoea. He had a fever, general weakness and headache but no chest pains, sore throat, leg swelling, paroxysmal nocturnal dyspnoea or orthopnoea. He had a recent travel history that suggested a COVID-19 exposure.

The man was in respiratory distress; oxygen saturation- 56% on room air, 82% on non-rebreather oxygen and his temperature was 37.6 °C. His blood pressure was 175/94 mmHg, and his pulse rate was 112 beats per minute regular. He had no pallor of mucous membranes, not jaundiced and hydration was fair. Examinations of the chest revealed reduced air entry in lung bases with crepitations. There were bronchial breath sounds over lower zones. Heart sounds I and II were present, normal and no murmurs were heard. No bi-pedal oedema was also noticed. The abdomen was soft, non-tender and there was no organomegaly. A diagnosis of bilateral pneumonia to rule out COVID-19 in a patient with diabetes and hypertension was made. Pulmonary embolism was a differential diagnosis. He was managed on intravenous fluids, anticoagulants, antibiotics, insulin and anti-hypertensive medications. He had a low haemoglobin (11.5g/dL) and a high white blood cell count (15.58 × 10^10^/L) with platelets at 185 × 10^10^/L. Liver and renal function tests were normal.

**FIGURE 4:**
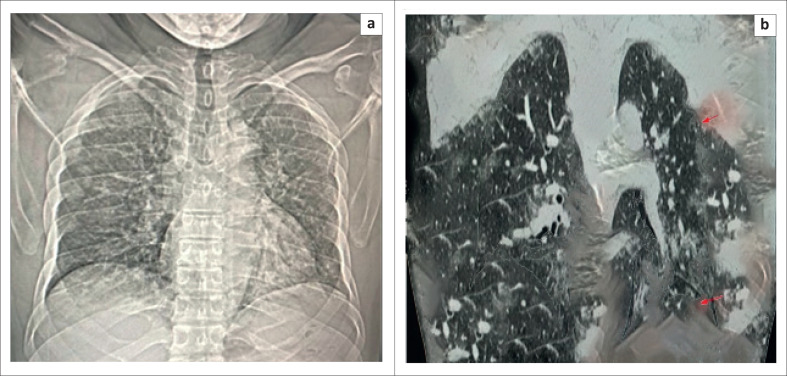
Radiological images of a 55 year old male patient with COVID-19 (37 Military Hospital, Accra, Ghana, May 2020). Case 3: (a) Postero-anterior chest x-ray showing bilateral ground-glass opacification and right upper and middle zone peripheral patchy opacities. (b) High resolution computerised tomography scan, coronal view, showing bilateral, ground-glass opacities with thickened interlobular and intralobular lines with ‘crazy paving’ appearance. Bilateral peripheral and subpleural opacities (red arrows).

A plain chest X-ray and a computerised tomography scan were requested (Figure 4). Later that day, his breathing became laboured and uneven. His temperature was 36.6 °C, pulse rate 100 beats per minute; respiratory rate was 29 per minute; oxygen saturation was 96% on intranasal oxygen. His blood pressure shot up to 220/140 mmHg but was controlled by intravenous labetalol.

A nasopharyngeal swab for SARS-CoV-2 testing was to be collected the following day but the patient passed before the sample could be taken. An academic autopsy was therefore ordered by the attending clinical team (Table 1).

**FIGURE 5:**
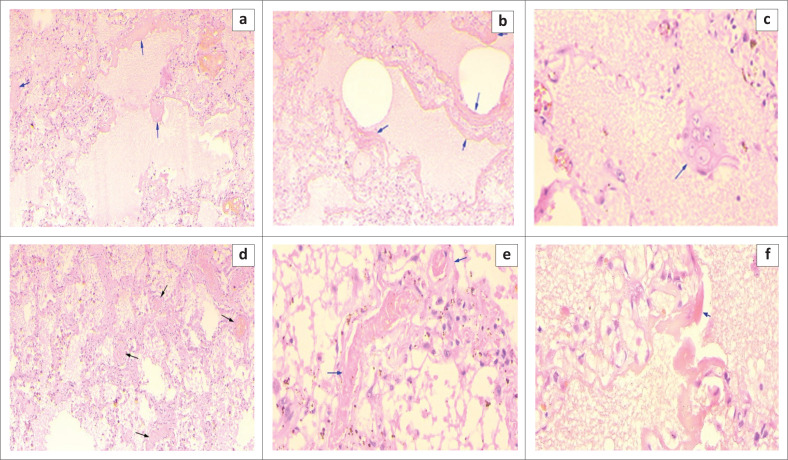
Haematoxylin and eosin staining of lung tissue samples from a 55 year old male patient with COVID-19 (37 Military Hospital, Accra, Ghana, May 2020). Case 3: (a) Severe pulmonary oedema, diffuse alveolar damage with hyaline membrane formation (blue arrow) ×100. (b) Severe pulmonary oedema, diffuse alveolar damage with hyaline membrane formation (blue arrow) ×100. (c) Large pneumocytes (blue arrow) ×400. (d) Microthrombi in small pulmonary arteries (black arrows) ×100. (e) Higher magnification (x400) of image (d) showing microthrombi (blue arrow) ×100. (f) Interstitial widening, pulmonary oedema and prominent hyaline membranes (blue arrow).

Postmortem bilateral lung parenchymal swabs were positive for SARS-CoV-2. Microscopic examination of the lungs showed severe congestion with haemorrhages. There were severe pulmonary oedema and moderately dense macrophage exudate. Diffuse alveolar damage with hyaline membrane formation was striking, and large pneumocytes showing enlarged nuclei and granular amphophilic cytoplasm were present. Microthrombi were also noted in some smaller pulmonary capillaries (Figure 5). In the final autopsy report, the cause of death was listed as COVID-19 pneumonia with diabetes mellitus and hypertension as contributory.

## Discussion

Though thousands of COVID-19 cases have been reported in Ghana with more than 30 deaths, no autopsies performed on such cases have been published in the medical literature. Postmortem samples were taken for COVID–19 testing in two of the cases that were autopsied. Both lung parenchymal samples were positive for SARS-CoV-2. In two cases, an earlier test using a nasopharyngeal swab sample was negative. This suggests that there is the possibility of false-negative results and that there is a proportion of COVID-19 patients who, because of their false-negative test results, will be managed in health facilities without protective protocols. Similarly, for those that die without being tested or have false-negative test results, their remains will be handled without the necessary safety precautions. To ensure the safety of workers and prevent unconscious exposure, health facilities and funeral homes should ensure that adequate safety measures are put in place during this pandemic.

In one case, the antemortem nasopharyngeal sample was negative for SARS-CoV-2, whereas the postmortem bronchopulmonary sample was positive for SARS-CoV-2. Such false-negative errors for COVID-19 may be the result of pre-analytical activities, such as a poorly collected sample, wrong sample labelling, mislabeling, interference from medications and improper storage or transport.^11,13^ It is therefore essential that great emphasis is made in ensuring adherence to proper sample collection and processing protocols across the entire cascade of activities in the diagnosis and reporting of COVID-19.

Considering the inevitable occurrence of deaths within the global COVID-19 pandemic, it is crucial for anatomical pathologists, while following due safety protocols, to look out for evidence of COVID-19 in all autopsy cases. The most significant pathological findings in our patients were in the lungs. This finding is similar to previous findings in other COVID-19 autopsies. In our patients, significant pathological findings that were present in all three patients included severe oedema, congestion, haemorrhages, the proliferation of pneumocytes, scattered multinucleated giant cells and DAD with hyaline membrane formation.^3,8,14^ Other pathological features including the proliferation of fibroblasts with interstitial fibrosis and microthrombi in the kidneys were seen in one patient.

Again, in a previous report, one patient had patchy inflammatory infiltrates that suggested a pneumonic process.^4^ Though all our patients had diabetes, we did not find the reported endotheliitis that has been reported to be present in some diabetic individuals.

Although molecular testing for Case 1 was not done, DAD was observed. This combined with the anecdotal evidence – contact with a COVID-19 patient and a later positive COVID-19 test of his caretaker – is suggestive of COVID-19. The death was thus ascribed to COVID-19 infection, in line with WHO^6^ Situation Report 94 for certifying deaths due to COVID-19 which states that:

[*W*]here a definite diagnosis of COVID-19 cannot be made but it is suspected or likely (e.g. the circumstances are compelling within a reasonable degree of certainty), it is acceptable to report COVID-19 on a death certificate as ‘probable’ or ‘presumed’. (p. 12)

The pathological presentation observed in the lungs were consistent with similar cases reported in Beijing and Oklahoma by Barton *et al.*,^7^ and Xu *et al*.^14^

### Limitations

Bronchopulmonary sampling was not done for SARS-CoV-2 testing for Case 1 as a result of the psychological unpreparedness of the autopsy team for COVID-19 autopsies. Additionally, no immunohistochemical staining was done for histopathological samples collected to show the presence and distribution of immune cells in the lungs. Also, the quality of photomicrographs could have been better if the bodies were better preserved after death.

### Conclusion

Findings from this study indicate that autopsies are capable of reporting evidence of COVID-19 and provides insights for proper sample management for purposes of diagnosis. More so, the diagnosis of COVID-19 at autopsy is relevant in situations where molecular testing such as rRT-PCR is not available to inform relevant public health action. Bronchopulmonary samples for SARS-CoV-2 testing during postmortem should be taken for presumed COVID-19 cases. More autopsies are required to fully understand the pathogenesis of this disease in Ghanaians.
